# Trends in Income Inequities in Cardiovascular Health Among US Adults, 1988–2018

**DOI:** 10.1161/CIRCOUTCOMES.123.010111

**Published:** 2024-04-03

**Authors:** Nicholas K. Brownell, Boback Ziaeian, Nicholas J. Jackson, Adam K. Richards

**Affiliations:** Division of Cardiology (N.K.B.), University of California, Los Angeles; Division of Cardiology (B.Z.), University of California, Los Angeles; Division of General Internal Medicine and Health Services Research (N.J.J.), University of California, Los Angeles.; Department of Global Health, Milken Institute School of Public Health, The George Washington University, Washington (A.K.R.).

**Keywords:** cardiovascular diseases, cross-sectional studies, nutrition surveys, poverty, socioeconomic factors

## Abstract

**BACKGROUND:**

Mean cardiovascular health has improved over the past several decades in the United States, but it is unclear whether the benefit is shared equitably. This study examined 30-year trends in cardiovascular health using a suite of income equity metrics to provide a comprehensive picture of cardiovascular income equity.

**METHODS:**

The study evaluated data from the 1988–2018 National Health and Nutrition Examination Survey. Survey groupings were stratified by poverty-to-income ratio (PIR) category, and the mean predicted 10-year risk of a major cardiovascular event or death based on the pooled cohort equations (PCE) was calculated (10-year PCE risk). Equity metrics including the relative and absolute concentration indices and the achievement index—metrics that assess both the prevalence and the distribution of a health measure across different socioeconomic categories—were calculated.

**RESULTS:**

A total of 26 633 participants aged 40 to 75 years were included (mean age, 53.0–55.5 years; women, 51.9%–53.0%). From 1988–1994 to 2015–2018, the mean 10-year PCE risk improved from 7.8% to 6.4% (*P*<0.05). The improvement was limited to the 2 highest income categories (10-year PCE risk for PIR 5: 7.7%–5.1%, *P*<0.05; PIR 3–4.99: 7.6%–6.1%, *P*<0.05). The 10-year PCE risk for the lowest income category (PIR <1) did not significantly change (8.1%–8.7%). In 1988–1994, the 10-year PCE risk for PIR <1 was 6% higher than PIR 5; by 2015–2018, this relative inequity increased to 70% (*P*<0.05). When using metrics that account for all income categories, the achievement index improved (8.0%–7.1%, *P*<0.05); however, the achievement index was consistently higher than the mean 10-year PCE risk, indicating the poor persistently had a greater share of adverse health.

**CONCLUSIONS:**

In this serial cross-sectional survey of US adults spanning 30 years, the population’s mean 10-year PCE risk improved, but the improvement was not felt equally across the income spectrum.

**WHAT IS KNOWN**

- National-level trends suggest improvement in mean cardiovascular disease burden in the United States, but persistent disparities exist when stratified by social determinants of health.
- Analyses of income-related disparities that compare the extremes of income distribution have shown persistent inequity in cardiovascular risk factors and outcomes.

**WHAT THE STUDY ADDS**

- In this serial cross-sectional nationally representative study of 26 633 participants from 1988 to 2018, there was improvement in the US population’s mean age- and sex-adjusted estimated 10-year risk of atherosclerotic cardiovascular disease.
- However, health equity metrics that account for the distribution of income across all strata show that income inequities persisted and even worsened over the same 30-year period, with much of the health benefit experienced by the wealthiest individuals in the United States.
- From 1988 to 2018, improvement in the mean cardiovascular health of the US adult population was not matched by improvement in cardiovascular income equity.

**See Editorial by Dixon and Sanchez**

Cardiovascular disease (CVD) is the leading cause of death in the United States^1^ and provides a unique opportunity to track progress toward the complementary public health goals of improving health and reducing health inequity.^2,3^ The dramatic decline in CVD burden represents one of the most impressive accomplishments in the history of medicine and public health in the United States. Age-adjusted CVD mortality declined by 60% between the 1980s and 2016, with approximately half of the reduction attributable to improvements in CVD treatment and half of the reduction attributable to risk factor mitigation.^4,5^

Despite the dramatic decline in CVD, evidence suggests that the rate of improvement may be slowing^6,7^ and that national-level trends may mask increasing inequities among subpopulations defined by sex, race and ethnicity, education level, and neighborhood disadvantage.^8–12^ Temporal analysis of CVD prevalence, risk factors, calculated risk scores, and mortality suggests that income-related CVD inequities are worsening^12–16^; however, an evaluation of socioeconomic inequities in CVD burden that simultaneously accounts for the decline in mean CVD risk over the past 3 decades has not been performed.

As recommended in the United States^17^ and abroad,^18^ health inequities should be reported on both relative and absolute scales,^19,20^ and comparisons of inequities between groups or different time periods should be placed in the context of overall (mean) health. To provide a full picture of equity, studies should use data for the entire population to inform priority setting for programs and policies. By performing relative and absolute comparisons between the 2 most extreme categories of an ordered variable, such as the highest and lowest income categories, information on a substantial portion of the population is ignored; furthermore, a decline in absolute risk across 2 groups can paradoxically increase relative measures of inequity, despite an improvement (risk reduction) in both groups. Due to the lack of consensus regarding the degree to which society should value health equity, recommendations are to present analyses using methods that accommodate multiple perspectives on the importance of reducing health inequity.^21^ This study applies a suite of health equity metrics to provide a comprehensive picture of CVD risk trends from 1988 to 2018, including those that do and do not account for the impact of income-related inequity. Utilizing data from the National Health and Nutrition Examination Survey (NHANES), we demonstrate how the narrative of success surrounding changes in CVD risk in the United States is tempered when society places an increasing value on ameliorating income inequities in cardiovascular health.

## METHODS

All data are publicly available at no cost from the National Center of Health Statistics (citation 23). NHANES is a repeated cross-sectional, nationally representative survey of the noninstitutionalized civilian US population and includes demographic, socioeconomic, health-related, and diet-related questions as well as laboratory-measured health factors. Since 1999–2000, NHANES has been released in 2-year cycles, with participants independently recruited for each cycle. We used data from NHANES III (1988–1994) and specific groupings from the continuous NHANES from 1999 to 2018, leading to 6 groupings in total: 1988–1994, 1999–2002, 2003–2006, 2007–2010, 2011–2014, and 2015–2018. For each survey cycle, the NHANES protocol was approved by the National Center for Health Statistics Research Ethics Review Board. The Institutional Review Board of the University of California, Los Angeles, determined that the research met the requirements for exempt international review board supervision.

### Data Collection

The analytic sample consisted of adults aged 40 to 75 years who participated in the household interview and physical examination portions of NHANES and whose 10-year CVD risk can be estimated by the pooled cohort equations (PCE).^22^ We excluded participants with a history of CVD.

### Demographic Characteristics and Cardiovascular Risk Factors

Self-reported data on sex, age, race and ethnicity, educational attainment, income, smoking status, and medication use for hypertension and diabetes were collected for each participant.^23^ Blood pressure, cholesterol, and glucose levels were directly measured. The poverty-to-income ratio (PIR) is a ratio of income to the contemporary poverty threshold and is comparable across survey years. A score greater (less) than 1 represents incomes above (below) the official poverty threshold.

### Outcomes

The primary health outcome was the predicted 10-year risk of a major cardiovascular event or death, as calculated by the PCE.^22^ Secondary health outcomes were CVD risk factors of diabetes, hypertension, hyperlipidemia, and current smoking as defined by Life’s Essential 8.^24^

Relative and absolute inequities were calculated in 2 complementary ways.^25^ First, relative (and absolute) inequities were defined as the ratio (and difference) of the mean PCE for the lowest PIR category (<1) and the mean PCE for the highest PIR category (5). A limitation of this approach is that only data from groups at the extremes of the income distribution contribute to the measure; participants whose income is between 100% and 500% of poverty are excluded from the calculation. When applied to the NHANES, this approach results in the exclusion of study participants, who represent more than half of the individuals eligible for primary prevention of CVD in the United States.

Second, we calculated summary equity metrics that allow all study participants to contribute to parameter estimates. Specifically, we calculated the relative concentration index (rCI), the absolute concentration index (aCI), and the achievement index.^26^

The concentration curve is a measure of relative inequity, derived by ranking individuals by a measure of socioeconomic standing (eg, income) on the *x* axis and plotting the cumulative share of health (eg, 10-year PCE risk) on the *y* axis. The rCI is then defined as twice the area between the concentration curve and the 45° diagonal. In a hypothetical, equal world where health is distributed evenly across income groups, the concentration curve would overlap with the diagonal 45° line, and the rCI would be 0. As relative inequity increases, the concentration curve strays from the diagonal, and the rCI increases in magnitude. The rCI can be defined by the following equation:


$$\begin{array}{l} rCI=\left(1-\frac{v}{n\mu }\overset{n}{\underset{i=1}{\sum }}y_{i}{\left(1-R_{i}\right)}^{v-1}\right) \end{array}$$

where $y_{i}$ is a measure of the *i*th person’s health based on PCE; μ is the mean of $y_{i}$; $R_{i}$ is the fractional rank of the individual according to income; and *v* is an inequity aversion parameter discussed in more detail below. A negative rCI indicates that adverse outcomes are concentrated among the poor; a positive rCI indicates that the rich have a greater share of adverse outcomes.

The aCI summarizes inequity on an absolute scale and is defined as the rCI multiplied by the mean level of health (μ, representing mean 10-year PCE risk):


$$\begin{array}{l} aCI=\mu *rCI \end{array}$$

In recognition that health policy aims to achieve reductions in both overall (mean) burden of CVD as well as to reduce inequities in the distribution of CVD, we calculated the achievement index as follows:


$$\begin{array}{l} AchievementIndex=\mu \left(1-rCI\right) \end{array}$$

The achievement index is an equity-weighted measure of CVD risk and describes the average risk (based on the PCE) in a population, accounting for the distribution of risk according to income. In the scenario of perfect equity, the rCI is 0 and the achievement index is μ. When the poor have a greater share of adverse outcomes, the rCI is negative and thus the achievement index >μ.

To accommodate alternative perspectives on the extent to which society should value ameliorating health inequity, the metrics used to calculate relative (rCI) and absolute (aCI) health inequities and the health achievement index incorporate the inequity aversion parameter *v.* Setting *v*=1 indicates that society is indifferent to inequity and that health achievement is judged exclusively by the mean level of health (rCI=0 and achievement index=μ=mean 10-year PCE risk); a large majority of health studies report only mean outcomes and are naive and implicitly agnostic to health inequities. The default value for *v* is 2, which translates into the health share of the poorest person weighted by a number close to 2, with weights declining in stepwise fashion; the health share of the median person is weighted as 1, and the health share of the richest person is weighted close to 0.^27^ Increasing values for *v* indicate society places greater value on addressing inequity; at the limit (*v*=∞), society would value the health of only the poorest members of society. Empirical studies suggest that similar to most normative values, society harbors a range of perspectives on health equity; it is unlikely that society will achieve consensus on a single value of *v* applicable to all settings.^28^ However, most individuals value equity in health outcomes to some degree, which suggests that *v*>1 for most outcomes. The inequity aversion parameter allows researchers, policymakers, and other stakeholders with diverse attitudes toward health equity to specify a range of values for *v* and to explore the potential implications of alternative equity weights on their conclusions.

For a specific value of *v*, an achievement plane can be used to display mean (average) health on the *x* axis and health equity on the *y* axis, oriented such that improvements in both overall health and the distribution of overall health are in the lower right (southeast) quadrant. Further descriptions of achievement planes can be found in the Supplemental Methods.

### Statistical Analysis

The rCI, aCI, and achievement index were calculated as outlined above, accounting for the complex survey design of NHANES.^29^ Inverse probability weights were used to extrapolate results to the population of US adults eligible for primary prevention of CVD. Analyses accounted for secular trends in the age and sex distributions of the population by including age and sex in the rCI regression equation. Confidence intervals were calculated by applying replicate survey weights to 10 000 bootstrap samples.^29,30^ All analyses were performed using Stata (College Station, TX).

## RESULTS

Of the 114 471 individuals who participated in the interview and physical examination portions of NHANES, we excluded 77 589 individuals younger or older than the age range (40–75 years) and 4493 individuals with a history of CVD. Information on specific variables related to the PCE or income status was missing for 5756 individuals; thus, 26 633 participants contributed to analyses (Figure S1). Participant characteristics are available in Table S1.

### Primary Outcome

**Figure 1.**
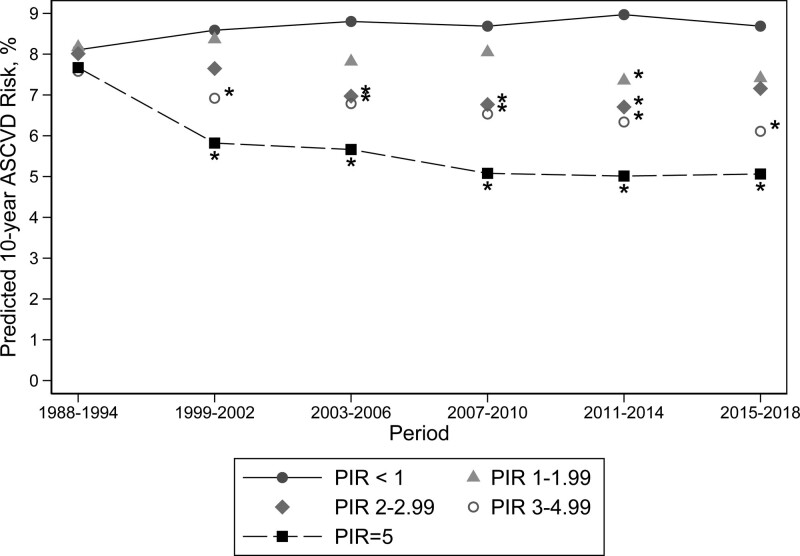
**Cardiovascular risk as calculated by the pooled cohort equations^22^ by poverty-to-income ratio (PIR) category, 1988–2018.** Atherosclerotic cardiovascular disease (ASCVD) risk is adjusted for age and sex. PIR is a ratio of income to the contemporary poverty threshold, where a score of 1 represents the official poverty threshold, a score >1 represents incomes above the official poverty threshold, and a score <1 represents incomes below the poverty threshold. *Significantly different from 1988–1994 (*P*<0.05) within the PIR group.

Among US adults, age- and sex-adjusted 10-year CVD risk predicted by the PCE improved from 7.8% in 1988–1994 to 6.4% in 2015–2018 (*P*<0.05; Table). Benefits were distributed inequitably according to income, with improvement over the 30-year period limited to the 2 highest income groups. Among individuals from households that reported PIR 5, PCE risk improved from 7.7% to 5.1%; in the PIR 3 to 4.99 group, PCE risk improved from 7.6% to 6.1% (*P*<0.05 for both comparisons). In contrast, PCE risk in the lowest income category (PIR <1) did not significantly change between 1988–1994 and 2015–2018 (8.1% to 8.7%; Figure 1).

**Table.**
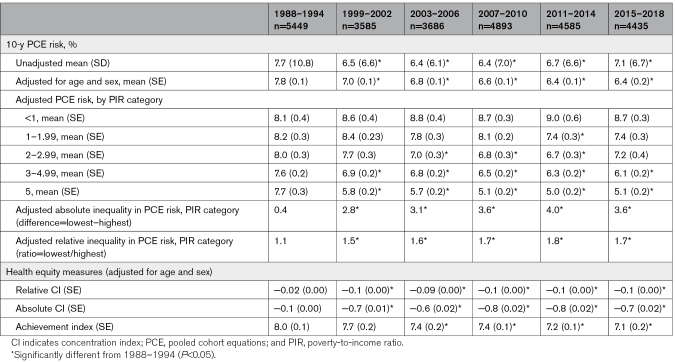
Trends in Select Health Equity Measures of Cardiovascular Risk as Calculated by the Pooled Cohort Equations,^22^ 1988–2018

### Inequity Measures

Income-related inequity in PCE risk increased substantially over the study period on both the relative and absolute scales. Using only the lowest and highest PIR categories, relative inequity (the ratio between mean PCE risk in the lowest and highest PIR categories) was negligible (1.1) in 1988–1994; it increased to 1.5 in 1999–2002 and reached 1.7 in 2015–2018 (*P*<0.05 for all subsequent time periods, compared with 1988–1994). That is, in 2015–2018, the predicted risk of a CVD event was 70% higher among individuals from households with income at or below the poverty level, compared with individuals from households with income ≥5× the poverty level, adjusting for differences in age and sex. Absolute inequity (the difference between mean PCE risk in the lowest and highest PIR categories) also increased, from 0.4% in 1988–1994 to 2.8% in 1999–2002 and 3.6% in 2015–2018 (*P*<0.05 for all subsequent time periods, compared with 1988–1994); this represents more than an 8-fold increase in absolute inequity.

**Figure 2.**
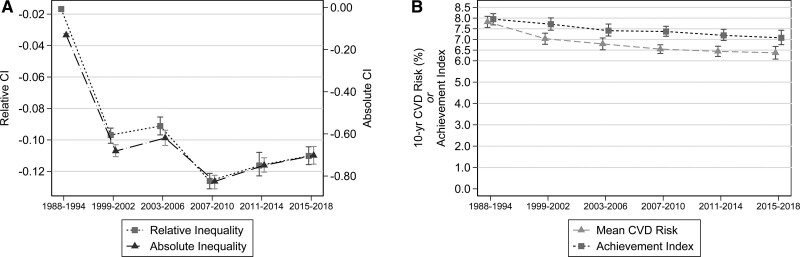
**Trends in relative and absolute inequity in predicted cardiovascular risk and health achievement, 1988–2018.** The relative concentration index (rCI), absolute concentration index (aCI), and achievement index are measures of health equity. **A**, The rCI is 0 when health is distributed evenly across income groups. A positive rCI indicates that adverse outcomes are concentrated among the wealthy; a negative rCI indicates that adverse outcomes are concentrated among the poor. The aCI summarizes inequity on an absolute scale, defined as the rCI multiplied by the mean level of health (mean cardiovascular disease [CVD] risk). **B**, The achievement index is an equity-weighted measure of mean CVD risk; the achievement index describes the mean CVD risk, accounting for the distribution of risk according to income. CI indicates concentration index.

Similar trends were noted when results were calculated using the rCI and aCI, which use data on all individuals from the entire continuous distribution of household income. Compared with the period from 1988–1994, the magnitude of the rCI and aCI was significantly larger in all subsequent time periods (Figure 2A); by 2015–2018, the rCI worsened from −0.02 to −0.1 (*P*<0.05) and the aCI worsened from −0.1 to −0.7 (*P*<0.05).

The calculation of the achievement index allows us to consider the improvement in mean PCE risk and the worsening income inequity in concert. Results of calculating the achievement index when applying a standard, low value for the inequity aversion parameter (*v*=2) suggest that overall health achievement improved between 1988–1994 and 2015–2018 (equity-weighted PCE risk [achievement index] declined from 8.0% to 7.1%; *P*<0.05). However, the magnitude of improvement was substantially attenuated, compared with the larger improvement in mean PCE risk, which is naive to changes in income inequity (Figure 2B).

**Figure 3.**
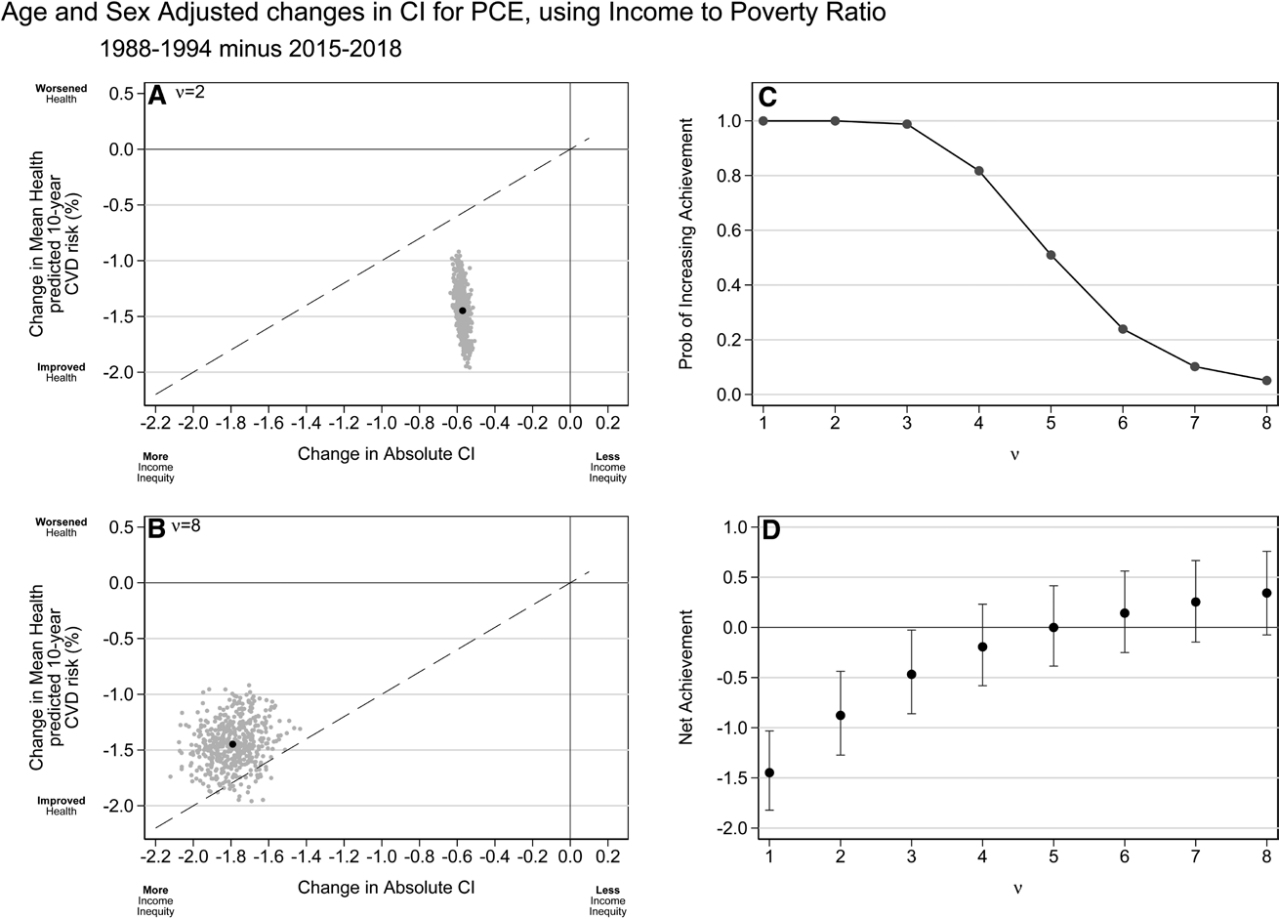
**Changes in cardiovascular risk inequities from 1988–1994 to 2015–2018, accounting for alternative attitudes toward the value of health equity.** Bootstrap replications for the incremental change in absolute inequity (as measured by the absolute concentration index [aCI]) and mean health (as measured by the age- and sex-adjusted pooled cohort equations [PCE]) from 1988–1994 to 2015–2018, where **A** represents a low aversion to inequity (inequity aversion parameter *v*=2) and **B** represents a high aversion to inequity (*v*=8). The point estimate (black dot) and its dispersion (gray dots) are shown on the graph. Points below the dotted line (*y*=*x*) indicate that the net improvement in health compensates for the net increase in inequity. **C**, Probability of an increase in achievement by the inequity aversion parameter. **D**, Net change in achievement index and 95% CIs for varying values of *v*. CVD indicates cardiovascular disease.

Figure 3A and 3B presents 2 achievement planes, each with the output of 1000 bootstrap replications calculating the temporal change in adjusted mean PCE risk (*y* axis) and the temporal change in absolute inequity (aCI; *x* axis). Point estimates are denoted with a dot. When *v*=2 (Figure 3A), society places a fairly low priority on addressing income inequity; *v*=8 (Figure 3B) indicates a high priority on addressing inequity. With regard to overall achievement, in Figure 3A (*v*=2), all (100%) bootstrap replications lie below the 45° line; this indicates a 100% probability that there is a temporal improvement (decrease) in income equity-weighted CVD risk when the inequity aversion is low (*v*=2) and that the worsening in income inequity over 30 years is compensated by the improvement in mean health. In contrast, when the inequity aversion is high (*v*=8; Figure 3B), almost none of the bootstrap replications lie below the 45° line, indicating a negligible probability of a temporal improvement in overall achievement. Figure 3C shows the proportions of bootstrap replications above the 45° line for different values of *v*, suggesting the probability of improving achievement at different inequity aversion parameter values; by *v*=5, there is a 50% probability of an improvement in achievement, and by *v*=7, there is a negligible probability of an improvement in achievement. Figure 3D shows the 95% CIs for the temporal change in achievement index at varying levels of *v*. When *v* is between 3 and 4, the net achievement is not significantly different from 0, indicating no change in achievement over 30 years. As *v* increases to 8, the point estimate increases above 0, indicating a worsening in achievement between the initial and final time periods; however, it is not significant at an α of 0.05. Recall that *v*=1 is the special situation where there is complete indifference to income inequity; in this situation, the net achievement is the difference in the mean PCE between 1988–1994 and 2015–2018 (an improvement of 1.5%).

### Cardiovascular Risk Factors

**Figure 4.**
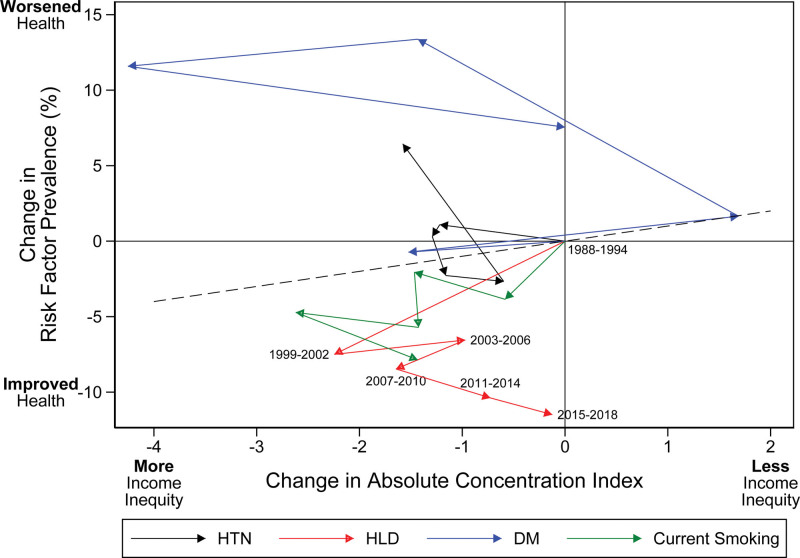
**Achievement plane displaying changes in health equity of cardiovascular risk factors, 1988–1994 to 2015–2018.** Achievement plane for cardiovascular risk factors (hypertension [HTN], hyperlipidemia [HLD], diabetes [DM], and current smoking) over a 30-year period. Change in health equity is presented on the *x* axis as the difference between the absolute concentration index (aCI) in each respective period compared with the aCI in 1988–1994. Calendar periods are labeled for HLD; periods are connected sequentially by vectors. For example, the southwest vector of HLD from 1988–1994 to 1999–2002 indicates that prevalence of HLD decreased and inequity increased.

Clinical variables that contribute to the PCE 10-year cardiovascular risk include hypertension, hyperlipidemia, diabetes, and smoking status. Changes in the prevalence and the absolute income inequity in these cardiovascular risk factors over 30 years in the United States are presented on an achievement plane (Figure 4): on the *x* axis is plotted the change in the aCI using a single value of the inequity aversion parameter (*v*=2); on the *y* axis is plotted the change in risk factor prevalence. Among the plotted risk factors, none are in the southeast (lower right) quadrant that would indicate reductions (improvements) in both prevalence and absolute income inequity. Two risk factors, smoking and hyperlipidemia, initially moved in a southwest direction on the achievement plane, representing decreased prevalence accompanied by increased absolute income inequity; more recently, they have moved in a southeasterly direction, representing unambiguous improvements in both prevalence and income inequity. In contrast, the most recent estimate for hypertension is located in the northwest quadrant, representing an unambiguous exacerbation of both prevalence and absolute income inequity since 1988–1994, with the most prominent northwesterly vector occurring since 2011–2014. Diabetes remains well above the dotted diagonal line, representing a worsening in health achievement since 1988–1994. This was driven by the large increase in diabetes prevalence; absolute income inequity in diabetes has been highly variable, though the most recent estimate is similar to that in 1988–1994. The achievement plane for changes in overall CVD risk is available (Figure S2).

## DISCUSSION

Based on NHANES data, the mean predicted 10-year cardiovascular risk in the United States has significantly improved over the past 30 years. However, such improvement is not equitably shared across the population; the use of multiple health equity metrics consistently demonstrates that inequities based on household income have worsened since 1988.

Previous studies have used NHANES to evaluate secular trends in cardiovascular risk factors and have documented persistent inequities based on socioeconomic status.^11–15^ Kanjilal et al^11^ found that between 1971 and 2002, the largest decline in the prevalence of cardiovascular risk factors occurred in the wealthiest income group. Subsequent studies^12–15^ used data from 1999 to 2018 to note that such disparities between the highest and lowest income groups persist; however, these studies have primarily used stratification by PIR to gauge income disparity. The use of a single metric to present equity can skew the interpretation of health information^28,31^; the current study complements prior work by utilizing a more comprehensive suite of equity metrics over a longer evaluation period.

Health inequities are most commonly reported on a relative scale, but best practice recommendations promote the additional use of an absolute scale as well as summary metrics to allow for varying weights of inequity.^17,31^ The PIR stratification provides an introduction to the persistent income disparity in the United States. Though mean cardiovascular health has improved over the past 30 years, the benefit continues to concentrate in the wealthiest PIR categories; the lowest PIR category has not experienced an improvement in cardiovascular health. As a result, when interpreting statistics that use data from only the highest and lowest PIR groups, absolute and relative income inequity worsened dramatically between 1988–1994 and 1999–2002 and have remained stagnant since.

Both overall health and health equity are valued, but there is a lack of consensus on how much society should value inequity reduction. Health equity metrics such as the rCI, aCI, achievement index, and achievement planes allow us to incorporate alternative attitudes toward the value of ameliorating health inequities.^26^ Furthermore, these metrics use all PIR categories available in NHANES. Like the relative and absolute income inequity measures, the rCI and aCI worsened between 1988–1994 and 1999–2002 and subsequently have not improved over the past 2 decades. When the inequity aversion parameter *v* is 2, the rCI can be interpreted as 1.3× the percentage of redistribution of CVD health from high- to low-income individuals (above and below median income, respectively) required to make income-related inequity equal to 0.^32^ Thus, to achieve an equal relative distribution of CVD risk in 2015–2018, the rCI of 0.1 suggests it would be necessary to redistribute 8.3% of CVD risk among individuals without CVD or ≈543 000 of the 6.6 million first CVD events predicted over the next 10 years.

Once this increase in relative inequity is considered by calculating an equity-weighted measure of cardiovascular achievement (the achievement index), the magnitude of the cardiovascular risk reduction is substantially attenuated. Compared with the decline in mean 10-year cardiovascular risk of 1.5% (from 7.8% to 6.4%), the inequity-weighted decline is 0.9% (achievement index from 8.0% to 7.1%), even when inequity aversion is low (*v*=2).

When individual cardiovascular risk factors that contribute to PCE risk (hypertension, hyperlipidemia, diabetes, and smoking status) are plotted on an achievement plane, none of the risk factors demonstrated an unequivocal improvement in health achievement over the 3-decade study period; that is, for none of the risk factors did both prevalence and income inequity decline. For diabetes and hypertension, prevalence and inequity increased over the 30-year time period. Our findings are consistent with prior evidence^12^; however, an evaluation of the primary drivers of changes in individual risk factors is beyond the scope of the present study.

An advantage of the methods used in the current study is the ability to vary the inequity aversion parameter *v*, which allows us to understand the degree to which society would need to value health equity to appreciate temporal improvements in mean cardiovascular health. As society’s aversion to inequity increases (*v* between 3 and 4), the perceived value of the reduction in mean 10-year PCE risk is offset by worsening income disparity. Clarke and Hayes^27^ evaluated secular changes in individual CVD risk factors in Australia and similarly found that a value *v* between 3 and 4 would offset gains in exercise between 1989 and 1995. Previous studies note that high-income societies care about reducing income-related health inequities; however, the extent to which they value reducing health inequity differs based on the population studied, the stakeholders responsible for prioritizing policy, the outcome of interest, and its distribution.^28,33–38^ Empirical studies that used the concentration index to elicit preferences for health equity-efficiency tradeoffs in Canada and Sweden^37,38^ found that the median values for the inequity aversion parameter *v* were between 1.5 and 3, though both noted substantial variation in the implicit values of inequity aversion between participants. In our study, lower income groups failed to experience a reduction in CVD risk, but we did not observe an increase in risk for any income group. This makes it unattractive to argue that the distribution of CVD risk in 1988 was preferable to the current distribution, but the fact that a fairly modest aversion to income inequity is sufficient to reduce or eliminate secular improvements in the achievement index suggests that the remarkable reduction in overall cardiovascular risk may not deserve such widespread celebration, as the country failed to simultaneously achieve the complementary goal of reducing cardiovascular income inequities.

SARS-CoV2 exacerbated inequities for many health outcomes, and future NHANES data collected during and after the pandemic may demonstrate an absolute increase in CVD risk in ≥1 income groups, in which case our methods provide a powerful tool to simultaneously consider changes in both income inequity and overall CVD risk. The equity metrics and visualizations can also be used to compare trends in CVD risk across states and regions and track changes in other health outcomes such as mortality, which has increased among non-Hispanic whites with lower educational attainment.^39^ Although examples of CVD in the United States are lacking, these methods facilitate the evaluation of guidelines, interventions, and policies that aim for the southeast corner of the achievement plane by ameliorating CVD inequities while reducing overall CVD risk.^40,41^ For example, Griffin et al^42^ evaluated 134 interventions included in the UK National Institute for Health and Care Excellence guidelines and found that half (52%) reduced inequity and improved health, one-third (32%) reduced health and increased inequity, and 16% involved a tradeoff between health and equity. When they ranked guidelines according to their impact on health and inequity, 12 of 30 (40%) were sensitive to the values of the inequity aversion parameter.

### Limitations

This study has several limitations. First, because NHANES is a series of consecutive cross-sectional surveys and not a panel study, individual-level assessment of changes in 10-year cardiovascular risk was not possible. Second, our study includes data through 2018 and may not reflect the contemporary distribution in 2024. Although additional NHANES data are publicly available (for 2019–2020), we chose to exclude data from a period that would only partially overlap with the SARS-CoV2 pandemic, when utilization declined inequitably for many health services.^43^ Third, our focus on a population eligible for primary prevention does not account for known inequities in the distribution of cardiovascular risk and risk factors among individuals who have already experienced a cardiovascular event.^44^ Fourth, the PCE 10-year cardiovascular risk calculator has not been validated in Hispanic or Asian populations and may overestimate the risk of CVD.^45^ Fifth, our study focused on cardiovascular risk factors included in the PCE and did not include other CVD risk factors with evidence for an independent association with CVD risk, including coronary artery calcium, C-reactive protein, and family history.^46^ Many of these risk factors are associated with socioeconomic status, and their absence from the PCE likely caused us to under-estimated income-related inequities in CVD risk.^47,48^ CVD risk equations in the United States should incorporate socioeconomic status measures such as educational attainment or neighborhood deprivation, as they do in other high-income countries.^48–50^ Sixth, our primary focus was income inequity; as a result, the current work did not address interactions with other known factors related to CVD equity, including structural racism, systemic bias, and housing instability. Limitations associated with the health equity metrics presented in this study can be found in the Supplemental Methods.

### Conclusions

The improvement in mean cardiovascular risk in the United States has not been complemented by a reduction in income inequities. Health inequities are multidimensional and complex and should be interpreted using a suite of health equity metrics that accommodate alternative value judgments about income-related variation in health. Our study shows that across numerous health equity metrics, cardiovascular income inequity has persisted or worsened since 1988. Public health practitioners and policymakers require novel approaches to achieve cardiovascular equity in the United States.

## ARTICLE INFORMATION

### Sources of Funding

Dr Richards was supported by a grant from the National Institutes of Health/National Institute of Neurological Disorders and Stroke (U54NS081764); statistical programming (by Dr Jackson) was supported by the Tides Foundation (TFR15-00194). Dr Brownell was supported, in part, by the Ruth L. Kirschstein National Research Service Award Institutional Research Training grant (5T32HL007895).

### Disclosures

All authors have not published, posted, or submitted any related papers from the same study. Dr Richards is an advisor to a fund at the Tides Foundation; he did not receive compensation from the Tides Foundation for this or any other study. The other authors report no conflicts.

### Supplemental Material

Supplemental Methods

Figures S1 and S2

Table S1

## Supplementary Material


