# Supplementary material for: Trends in Income Inequities in Cardiovascular Health Among US Adults, 1988–2018
Source: Circ Cardiovasc Qual Outcomes. 2024 Apr 3;17(5):e010111. doi: 10.1161/CIRCOUTCOMES.123.010111 (PMC11104495; doi:10.1161/CIRCOUTCOMES.123.010111)
Supplement: Supplementary file 1 [file hcq-17-e010111-s001.pdf]

SUPPLEMENTAL MATERIAL

Supplemental Methods

Achievement Planes

An achievement plane can be used to display temporal changes in both overall levels of health and in how equitably they are distributed across the population. The x-axis shows the difference between mean level of ill-health (i.e. change in mean 10-year PCE risk) relative to a baseline time period, while the y-axis shows the change in absolute inequity (i.e. change in absolute concentration index) between the same points in time. The southeast quadrant of the achievement plane indicates the most desirable region where there has been a decline in both ill-health and absolute health inequity; the northwest quadrant represents the least desirable region where both ill-health and health inequity have increased. In the other quadrants, there is a tradeoff between change in mean ill-health and change in inequity. Extending a 45-degree line through the southwest quadrant, points below the line would indicate that the net improvement in health compensates for the net increase in inequity. In contrast, points above the line would indicate that the net increase in inequity is greater than the net improvement in health, suggesting an overall increase in the achievement index for an adverse health outcome such as CVD risk.

Limitations of Health Equity Metrics

The health equity metrics used in this study should be interpreted together, considering their respective strengths and limitations. First, these health equity metrics are inherently insensitive to changes in socioeconomic position that do not impact socioeconomic ranking; thus, an

increase in the absolute level of income among the poor would not change socioeconomic ranking, and would not change the relative concentration index, if all income groups experience a similar increase in absolute income. Second, although a smaller value of the rCI or the achievement index typically suggests an improvement (reduction) in health inequity, it is possible for the rCI and achievement index to decline as a result of a worsening in health among the rich, even if overall (mean) health of the population does not improve.[35] This hypothetical change is rarely documented in studies of health inequity and if it were, then the seemingly counter-intuitive result would not be unique to the equity metrics presented here; it also is a feature of simpler ratio- and difference-based measures. The hypothetical possibility highlights the importance of considering measures of both relative and absolute inequity, and to do so in concert with measures of overall (mean) health in the population. Third, the rCI, aCI, and achievement index are not the only measures of health inequity.[17,35] The current suite was chosen to provide a broad range of metrics, from the simple (e.g., relative income inequality) to the more comprehensive (e.g., achievement index); including all available equity metrics is outside the scope of this study.

Supplemental Figures and Figure Legends

Figure S1: Flowchart of study participants

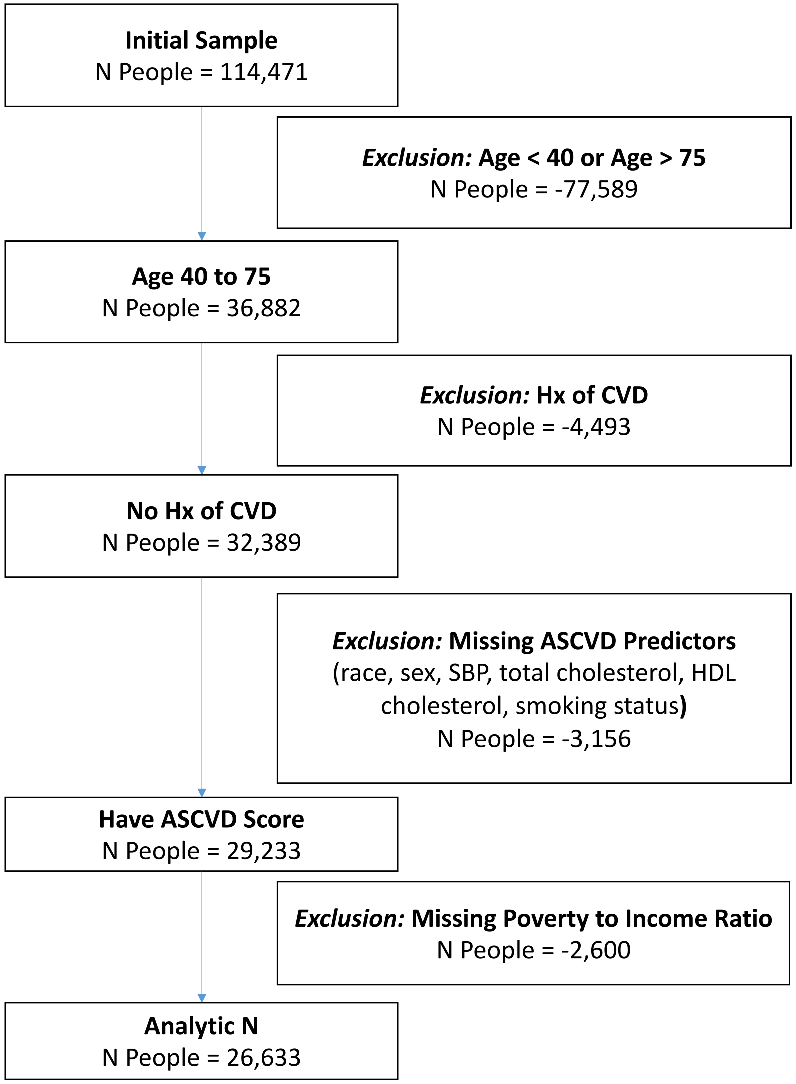

Figure S2: Achievement Plane Displaying Changes in Equity Based on Cardiovascular Risk, 1988-1994 to 2015-2018

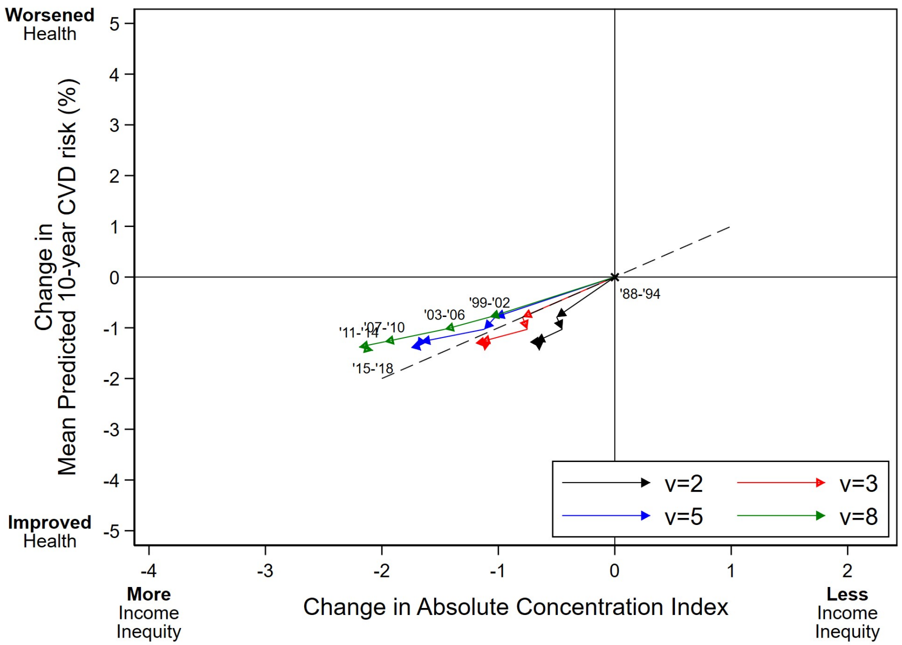

Inequity is presented on the x-axis as the change in the aCI in each respective calendar period compared to the aCI in 1988-1994; on the y-axis is plotted the change in mean PCE 10-year CVD risk. Coordinates in the lower right (southeast) quadrant represent an unambiguous improvement in (lowering of) both inequity and mean health; coordinates in the upper left (northwest) quadrant represent an unambiguous worsening (increase in) both inequity and mean health. Coordinates in the southwest and northeast quadrants represent tradeoffs between changes in mean health and changes in absolute inequity; the dotted diagonal line represents points where changes in mean health and absolute inequity are equivalent in magnitude. Alternative values for the inequity aversion parameters are graphed. When $v = 2$, health achievement for cardiovascular risk has improved since 1988-1994 (i.e., line lies to the right of the 45-degree line). However, the improvement in mean health is accompanied by a temporal worsening of inequity; when $v = 5$, the increase in absolute inequity is similar to the improvement in mean health (i.e., point estimates lie slightly left of the 45-degree line). When $v = 8$, the distance from the diagonal dotted line gradually increases in a southwesterly direction, providing a visual representation of how improvements in mean CVD risk are more than offset by respective increases in absolute inequity.

Supplemental Tables

Table S1: Demographics of Study Participants in the National Health and Nutrition Examination Surveys, 1988-2018

	Subgroup	**1988-1994** N=5,449	**1999-2002** N=3,585	**2003-2006** N=3,686	**2007-2010** N=4,893	**2011-2014** N=4,585	**2015-2018** N=4,435
US Population Represented		61,442,929	80,172,075	90,353,398	94,490,966	101,672,220	103,325,878
Age (mean ± SE)	Years	53.91 ± 11.43	53.02 ± 7.65	53.22 ± 7.16	53.87 ± 7.90	54.56 ± 7.42	55.54 ± 7.35
Sex*	Female	52.1%	52.2%	51.9%	52.4%	51.9%	53.0%
	Male	47.9%	47.8%	48.1%	47.6%	48.1%	47.0%
Race	White	87.3%	76.6%	77.1%	74.8%	71.7%	68.8%
	Black	9.1%	8.8%	9.7%	9.5%	10.0%	9.2%
	Mex-US Born	2.1%	2.2%	2.2%	2.4%	2.5%	2.9%
	Mex-Non US Born	1.6%	2.8%	3.4%	4.2%	3.8%	4.5%
	Other		9.7%	7.7%	9.2%	12.0%	14.6%
Poverty to Income Ratio (PIR) Category	<1	7.5%	9.8%	8.3%	10.1%	11.6%	10.2%
	1-2	16.3%	14.9%	15.8%	16.9%	17.4%	16.4%
	2-3	19.6%	14.4%	14.6%	14.5%	13.3%	13.8%
	3-5	32.7%	27.4%	29.9%	24.7%	25.7%	25.6%
	≥5	23.9%	33.6%	31.4%	33.9%	32.0%	34.0%
Educational Attainment	<9th Grade	10.4%	6.3%	6.0%	6.2%	4.8%	4.4%
	<12th Grade	12.9%	12.1%	8.7%	11.6%	9.4%	6.9%
	Highschool Grad	34.7%	24.5%	26.6%	24.1%	20.4%	22.4%
	Some College	17.7%	27.8%	31.3%	28.2%	31.0%	32.0%
	College Grad	24.2%	29.2%	27.5%	30.0%	34.4%	34.4%

Footnote:
* All subgroups significantly different by period (p<0.05) except for sex.
Note: NHANES III (1988-1994) did not have a code for Race-Other.
